# Gene Expression Profiling Identifies Important Genes Affected by R2 Compound Disrupting FAK and P53 Complex

**DOI:** 10.3390/cancers6010166

**Published:** 2014-01-21

**Authors:** Vita M. Golubovskaya, Baotran Ho, Jeffrey Conroy, Song Liu, Dan Wang, William G. Cance

**Affiliations:** 1 Department of Surgical Oncology, Roswell Park Cancer Institute, Buffalo, NY 14263, USA; E-Mails: baotran.ho@roswellpark.org (B.H.); william.cance@roswellpark.org (W.G.C.); 2 Genomics Shared Resource, Center for Personalized Medicine, Roswell Park Cancer Institute, Buffalo, NY 14263, USA; E-Mail: jeffrey.conroy@Roswellpark.org; 3 Bioinformatics Core Facility, Biostatistics, Roswell Park Cancer Institute, Buffalo, NY 14263, USA; E-Mails: song.liu@roswellpark.org (S.L.); dan.wang@roswellpark.org (D.W.)

**Keywords:** Focal Adhesion Kinase, p53, Mdm-2, Nutlin, gene expression profiling, microarrays, combination therapy

## Abstract

Focal Adhesion Kinase (FAK) is a non-receptor kinase that plays an important role in many cellular processes: adhesion, proliferation, invasion, angiogenesis, metastasis and survival. Recently, we have shown that Roslin 2 or R2 (1-benzyl-15,3,5,7-tetraazatricyclo[3.3.1.1~3,7~]decane) compound disrupts FAK and p53 proteins, activates p53 transcriptional activity, and blocks tumor growth. In this report we performed a microarray gene expression analysis of R2-treated HCT116 p53^+/+^ and p53^−/−^ cells and detected 1484 genes that were significantly up- or down-regulated (*p* < 0.05) in HCT116 p53^+/+^ cells but not in p53^−/−^ cells. Among up-regulated genes in HCT p53^+/+^ cells we detected critical p53 targets: Mdm-2, Noxa-1, and RIP1. Among down-regulated genes, Met, PLK2, KIF14, BIRC2 and other genes were identified. In addition, a combination of R2 compound with M13 compound that disrupts FAK and Mmd-2 complex or R2 and Nutlin-1 that disrupts Mdm-2 and p53 decreased clonogenicity of HCT116 p53^+/+^ colon cancer cells more significantly than each agent alone in a p53-dependent manner. Thus, the report detects gene expression profile in response to R2 treatment and demonstrates that the combination of drugs targeting FAK, Mdm-2, and p53 can be a novel therapy approach.

## 1. Introduction

Focal Adhesion Kinase (FAK) regulates many important cellular processes: proliferation, adhesion, spreading, motility, and survival [1,2,3]. The previous study demonstrated that p53 bound FAK promoter and repressed FAK transcription [4,5]. In addition, an analysis of 600 breast cancer tumors demonstrated that mutations of p53 highly correlated with FAK overexpression [6,7]. Moreover, estradiol caused p53-dependent inhibition of FAK expression in breast cancer cells [8]. These studies demonstrated the association of FAK and p53 signaling pathways in cancer [4,9].

Recently we have demonstrated a direct interaction of p53 protein with the *N*-terminal domain of FAK [4,10]. The interaction of FAK and p53 has been confirmed by another group, who demonstrated that FAK interacts not only with p53 but also with Mdm-2 to down-regulate p53 by ubiquitination [11]. We identified the 7 amino-acid binding site (amino-acids 65-72) in the proline-rich region of p53 protein that is involved in interaction with FAK [12] and by *in silico* modeling of NCI compound database we identified a small molecule compound Roslin 2 or R2 (1-benzyl-15,3,5,7-tetraazatricyclo[3.3.1.1~3,7~]decane) that directly bound to the *N*-terminal domain of FAK and disrupted FAK and p53 complex [13]. The R2 compound decreased viability and colony formation of colon cancer cells in a p53-dependent manner, and reactivated transcriptional activity of p53 with p21, Mdm-2 and Bax transcriptional targets [13]. In this report we performed microarray analysis with Illumina HumanRef-8 v3 bead chip on untreated HCTp53^+/+^ and HCTp53^−/−^ cells and on the cells treated with R2 compound and revealed >1,484 genes significantly up- or down-regulated by R2 compound in a p53-dependent manner (*p* < 0.05). Among these genes the known p53 pro-apoptotic targets, such as Mdm-2, Noxa-1, and RIP1, were detected. Moreover, the combination of R2 with either M13 disrupting FAK and Mdm-2, or with Nutlin-1 disrupting p53 and Mdm-2 interaction, decreased HCT116 cancer cell clonogenicity more efficiently than each inhibitor alone in a p53-dependent manner. Thus, this study revealed a gene expression profile in response to R2 and demonstrated that its combination with M13 or Nutlin-1 can be a novel therapy approach to reactivate p53 and decrease the survival of cancer cells.

## 2. Results

### 2.1. Global Microarray Gene Profiling in HCT116 Cells Treated with R2 Demonstrated Up- and Down-Regulates Genes in a p53-Dependent Manner

**Figure 1:**
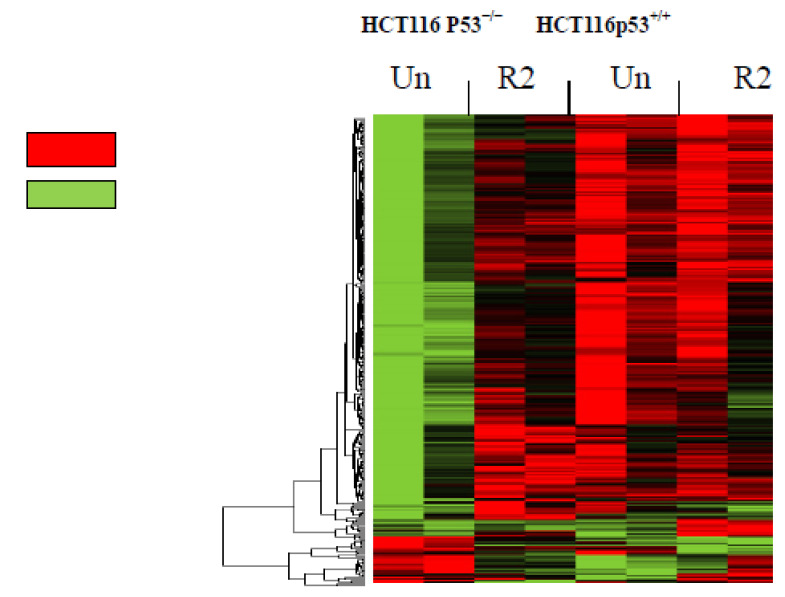
Gene expression profiling in HCT116 p53^+/+^ and HCT116 p53^−/−^ cells in response to R2. The heatmap of untreated and R2-treated cells demonstrated significantly up-and down-regulated (*p* < 0.05) genes in colon cancer cells. The heatmap is shown in the left panel. Red indicates significantly up-regulated genes, and green indicates down-regulated genes (*p* < 0.05). Un-marks Untreated cells, R2 marks HCT116 cells treated with 25 μM R2 for 24 hours.

To detect global gene profiling and reveal differentially expressed genes in response to R2 compound that disrupted FAK and p53 interaction [13], we performed a microarray analysis in R2-treated and untreated HCT116 p53^+/+^ and HCT116 p53^−/−^ cells using an Illumina Human ref-8 v3.0 bead chip and compared global gene expression (Figure 1). Since recently we detected that treatment of HCT116 cells at 25 μM of R2 for 24 hours significantly up-regulated transcriptional activity of p53, we treated HCT116 p53^+/+^ and HCT116p53^−/−^ cells with this dose of compound for 24 hours. The heatmap of the genes that are significantly up- or down-regulated, *p* < 0.05 in untreated and R2-treated HCT116 p53^+/+^ and p53^−/−^ cells is shown in Figure 1. We detected 1,484 genes that were differentially expressed: down or up-regulated by 25 μM R2 treatment for 24 hours in HCT116 p53^+/+^ cells but not in HCT116 p53^−/−^ cells (*p* < 0.05). The 961 genes were ≥1.2 fold significantly affected in HCT116p53^+/+^ cells, but not in HCTp53^−/−^ cells. The Table 1 presents a list of important genes ≥1.2 fold significantly up- or down-regulated in HCT116 p53^+/+^ cells (*p* < 0.05), but not in HCT116 p53^−/−^ cells and also shows several genes that were significantly inversely affected in both cell lines (marked by an asterisk).

Among the genes up or down-regulated by R2 in a p53-dependent manner, we detected up-regulated targets of p53, such as Mdm-2 and Noxa1 and pro-apoptotic proteins RIP-1 (Table 1). Among the down-regulated genes Met oncogene, PLK2, MTF1, KIF14, BIRC2 and other important genes were detected (Table 1).

**Table 1:** Several up-regulated and down-regulated genes by 25 μM R2 in HCT116 p53^+/+^ cells in a p53-dependent manner (≥1.2-fold, *p* < 0.05).

Up-regulated genes													
						HCT116 p53^+/+^ cells				HCT116p53^−/−^ cells			
**Entrez** **Gene ID**		**Gene Symbol**	**Name**		**Function**	**Fold Change** **R2-treated/Untreated**		***p*-value**		**Fold Change** **R2-treated/Untreated**		***p*-value**	
**8737**		**RIPK1**	**Receptor (TNFRSF)-interacting serine-threonine kinase 1**		**Apoptosis, regulation of I-kappa B/NF-kappa B cascade**	**1.20**		**0.01**		**0.95**		**0.46**	
3725		JUN	Jun oncogene Serine-threonine kinase 1		Transcription	1.21		0.07		1.01		0.83	
**10811**		**NOXA1**	**NADPH oxidase activator**		**Superoxide metabolic process**	**1.20**		**0.01**		**0.85**		**0.02**	
8739		HRK	Harakiri, Bcl2 interacting protein		Induction of apoptosis	1.22		0.01		1.06		0.37	
**4193**		**MDM2**	**Mdm2 p53 binding protein homolog (mouse)**		**Ubiquitination, negative regulation of proliferation**	**1.22**		**0.03**		**0.92**		**0.29**	
10608		MXD4	MAX dimerization protein 4		Negative regulation of transcription	1.23		0.025		1.00		0.93	
5602		MAPK13	Mitogen-activated protein kinase 13		Protein phosphorylation	1.23		0.017		0.87		0.08	
3429		IFI27	Interferon inducible protein 27		Interferon-regulated process	1.25		0.013		0.99		0.93	
5300		PIN1	Peptidylprolil cis/trans isomerase		Cell cycle, protein folding	1.26		0.004		0.95		0.49	
**7159**		TP53BP2 *	**Tumor p53 binding protein 2**		**Apoptosis**	**1.28**		**0.014**		**0.79**		**0.02**	
9064		MAP3K6	Mitogen-activated kinase-kinase 6		Protein phosphorylation	1.28		0.003		1.08		0.22	
1396		CRIP1	Cysteine-rich protein 1		Cell proliferation	1.31		0.026		1.17		0.046	
7867		MAPKAPK3	Mitogen-activated protein kinase 3		Protein phosphorylation	1.36		0.0008		0.95		0.48	
3675		ITGA3	Integrin, alpha 3		Cell adhesion	1.40		0.0003		1.1		0.13	
5296		PIK3R2	Phosphoinositide-3-kinase, regulatory subunit		Signal transduction	1.41		0.00019		0.88		0.084	
83990		BRIP1 *	BRCA1 interacting protein *C*-terminal helicase 1		DNA damage checkpoijnt metabolic process	1.43		0.0024		0.64		0.00056	
90850		ZNF598	Zinc finger protein 598		Protein binding	1.51		0.0029		1.07		0.41	
3659		IRF1	Interferon regulatory factor 1		Regulation of interleukin-12	1.55		9.04E-05		1.00		0.99	
85441		PRIC285	Peroxisomal proliferator-activated receptor A interacting complex 285		Regulation of transcription	1.58		4.98E-05		0.83		0.028	
22937		SCAP	SREBF chaperone		Lipid metabolic process	1.61		1.5E-0.5		0.96		0.51	
5652		PRSS8	Protease, serine, 8		Proteolysis	1.81		2.21E-06		0.96		0.59	
147166		TRIM16L *	Tripartite motif-containing 16-like			1.84		0.008		0.47		0.0025	
**Down-regulated genes**													
							**HCT116 p53^+/+^ cells**				**HCT116p53^−/−^ cells**		
**Entrez** **Gene ID**	**Gene Symbol**		**Name**	**Function**			**Fold Change** **R2-treated/Untreated**		***p*-value**		**Fold Change** **R2-treated/Untreated**		***p*-value**
7272	TTK *		TTK protein kinase	Protein amino acid phosphorylation; Mitotic spindle organization			0.82		0.016		1.2		0.02
**10769**	**PLK2**		**Polo-like kinase 2**	**Mitotic cell cycle**			**0.82**		**0.019**		**1.05**		**0.47**
4233	MET		Met proto-oncogene	Protein amino acid phosphorylation; Multicellular organismal development			0.81		0.01		0.89		0.14
**27085**	**MTBP**		**Mdm-2, transformed 3T3 cell double minute 2, p53 binding protein**	**Ubiquitin-dependent protein catabolic process; cell cycle**			**0.80**		**0.0066**		**1.13**		**0.1**
4436	MSH2		Mut S homolog 2	Mismatch repair			0.8		0.017		0.88		0.13
2353	FOS		FOS oncogene	DNA methylation, transcription			0.77		0.032		1.07		0.51
64282	PAPD5 *		PAP associated domain 5	DNA replication; cell cycle			0.77		0.028		1.77		0.0007
9928	KIF14		Kinesin 14	Microtubule-based movement			0.76		0.01		1.02		0.82
9112	MTA1		Metastasis associated 1	Regulation of transcription			0.75		0.026		1.26		0.06
7013	TERF1 *		Telomeric repeat binding factor 1	Telomere maintenance via telomerase			0.74		0.001		1.26		0.0058
5099	PCDH7		Procadherin 7	Cell adhesion			0.73		0.002		1.16		0.09
4774	NFIA		Nuclear factor 1/A	DNA replication; transcription			0.72		0.008		1.23		0.06
54434	SSH1 *		Slingshot homolog 1	Cell morphogenesis; cytoskeleton			0.65		0.015		1.53		0.015
329	BIRC2		Baculoviaral IAP repeat-containing 2	Cell surface receptor linked signaling pathway; regulation of apoptosis			0.62		1.43E-05		0.93		0.36
4520	MTF 1 *		Metal regulatory transcription factor 1	Regulation of transcription; Response to oxidative stress			0.58		0.003		1.66		0.004

The genes encoding p53 targeted proteins or proteins associated with p53 pathway are marked in bold. * The significantly inversely affected genes in HCT116 p53^+/+^ and HCT116 p53^−/−^ marked with an asterisk.

**Figure 2:**
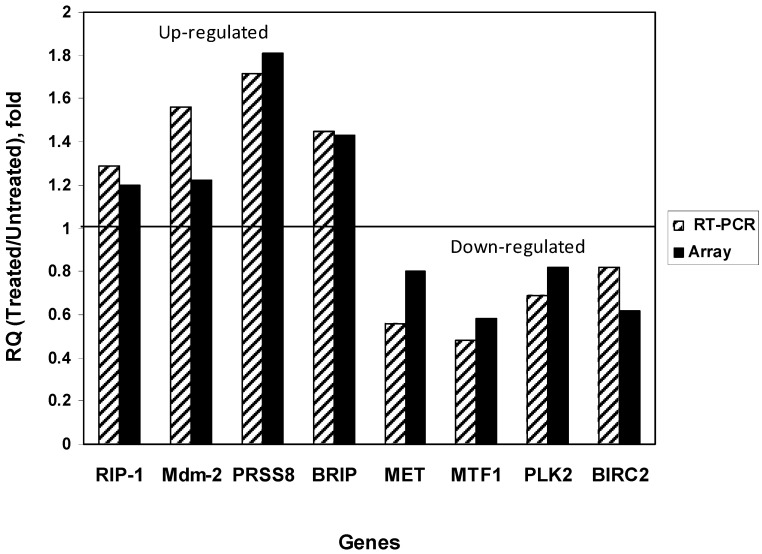
Real-time confirmed and validated differentially expressed up- and down-regulated (*p* < 0.05) genes in R2-treated cells *versus* untreated cells. The fold of RQ treated to untreated ratios is shown for RT-PCR. The bold black bars show average fold of ratios of R2 treated to untreated by microarray analysis and the striped black and white bars show average fold of mRNA levels of gene expression by RT-PCR analysis. Up-regulated and down-regulated genes are marked. The changes are statistically significant, *p* < 0.05.

Real-time PCR analysis confirmed and validated several up-regulated genes: RIP1, Mdm-2, PRSS8, and BRIP, and several down-regulated genes: MET, MTF1, PLK1, and BIRC2 (Figure 2). The data of RT-PCR and microarray analyses were very similar (Figure 2). Thus, R2 reactivated p53 activity and affected many p53 target and critical genes in a p53-dependent manner.

### 2.2. The R2 Sensitized Cancer Cells to M13 (Disrupting FAK and Mdm-2) and Nutlin-1 (Disrupting p53 and Mdm-2) Treatments

**Figure 3:**
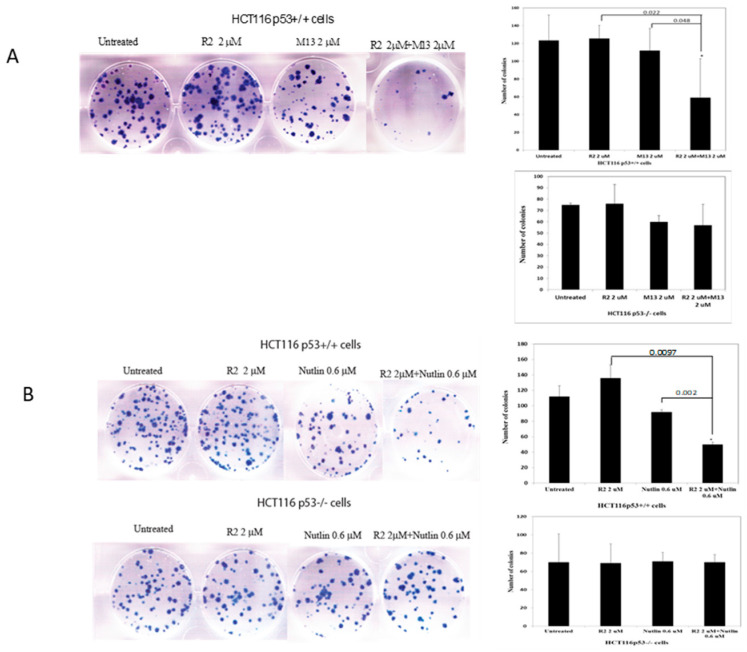
R2 sensitized HCT116 cells to M13 and Nutlin. A clonogenicity assay was performed on HCT116p53^+^/^+^ cells or HCT116p53^−^/^−^ treated for one week either with R2 alone, M13 alone, or with a combination of R2 and M13 (**A**), and R2 alone, Nutlin-1 alone, or with a combination of R2 and Nutlin-1 (**B**). The representative clonogenicity assay of several independent experiments is shown. A clonogenicity assay image of HCT116p53^−^/^−^ in (**A**) is not shown. Colonies were counted and quantification is shown with the panels on the right. Bars show the average number of colonies per treatment from two to four independent experiments. * Student’s *t*-test, *p* < 0.05, R2 + M13 or R2 + Nutlin-1 versus untreated HCT116p53^+/+^ and HCT116 p53^−/−^ or treated cells with a single agent. The *p*-values are shown in a combination group versus a single agent.

Since we detected p53-dependent up-regulation of Mdm-2 expression (Table 1) in HCT116p53^+/+^ cells, and Mdm-2 was shown to bind FAK to down-regulate p53, providing survival to cancer cells [11], we used compound M13 that disrupted interaction of FAK and Mdm-2, called M13 that up-regulated p53 activity and caused apoptosis in HCT116 p53^+/+^ cells [14] in combination with R2 in clonogenicity assay to test if combination of R2 and M13 will decrease cancer cell clonogenicity more effectively than each agent alone. Figure 3A shows that combination of R2 and M13 is more effective in decreasing clonogenicity of HCT116 p53^+/+^ cells than each agent alone. This effect was not observed in HCT116 p53^−/−^ cells.

Since Nutlin-1 that disrupts p53 and Mdm-2 protein interaction [15], also can induce p53, we treated HCT116 p53^+/+^ and HCT53^−/−^ cells with either R2 alone, Nutlin-1 alone or with combination of R2 and Nutlin-1 (Figure 3B). The combination of R2 and Nutlin-1 also decreased number of colonies compared with each agent alone in HCT116 p53^+/+^ cells in a p53-dependent manner (Figure 3B). Thus, the combination of R2 and M13 or R2 and Nutlin-1 was more effective in decreasing clonogenicity than with each agent alone in HCTp53^+/+^ cells, but not in HCT116 p53^−/−^ cells (Figure 3).

Thus, a combination of compounds that target FAK and p53, FAK and Mdm-2 or p53 and Mdm-2 can be an effective therapeutic approach to block colon cancer cells.

## 3. Experimental

### 3.1. Cell Lines and Culture

The early passage HCT116p53^−^/^−^ and HCT116p53^+^/^+^ colon cancer cells were obtained from Dr. Bert Vogelstein (Johns Hopkins University, Baltimore, MD, USA) and maintained in McCoy’s5A medium with 10% FBS and 1 μg/mL penicillin/streptomycin. The cells were passaged less than two month after resuscitation of frozen aliquots.

### 3.2. Reagents

R2 (1-benzyl-15,3,5,7-tetraazatricyclo[3.3.1.1~3,7~]decane) was kindly provided by Drs. Ethirajan Manivannan and Ravindra Pandey (Roswell Park Cancer Institute, Buffalo, NY, USA). M13 (5'-*O*-tritylthymidine) and Nutlin-1 were obtained from Sigma (St. Louis, MO, USA). The compounds were solubilized in DMSO at a concentration of 25 mM.

### 3.3. Clonogenicity Assay

One thousand cells were plated on six-well plates and incubated with different R2 compound, M13, Nutlin-1 or with combination of R2 and M13 or R2 and Nutlin-1 compounds or without compound for seven or ten days. Then cells were fixed in 25% methanol and stained with Crystal Violet. The colonies were counted from two of four independent experiments per each treatment group.

### 3.4. RNA Isolation

The untreated and treated cells with 25 μM R2 for 24 hours were used for isolation of RNA. Total RNA was isolated from the frozen cell pellets using the RNeasy midi kits (Qiagen, Inc., Valencia, CA, USA) following the manufacturer’s instructions. Before labeling, RNA samples were quantitated using a ND-1000 spectrophotometer (Nano Drop, Wilmington, DE, USA).

### 3.5. Microarray Analysis

Expression profiling was accomplished using the HumanRef-8 whole-genome gene expression array and direct hybridization assay (Illumina, Inc., San Diego, CA, USA). Initially, 500 ng total RNA was converted to cDNA, followed by *in vitro* transcription to generate biotin labeled cRNA using the Ambion Illumina Total Prep RNA Amplification Kit (Ambion, Inc., Foster City, CA, USA) according to the manufacturer’s instructions. The labeled probes were hybridized overnight at 58 °C to the Illumina HumanRef-8v3BeadChips. Following washing and staining with Cy3-streptavidin conjugate, the BeadChips were imaged using the Illumina Bead Array Reader to measure fluorescence intensity at each probe. The raw intensity of each Illumina Human ref-8 v3.0 gene expression array was scanned and extracted using Bead Scan, with the data corrected by background subtraction in Genome Studio (v2010.1), gene expression module (v1.6.0). The BeadChip data files were further analyzed with a *Bioconductor* package to determine gene expression signal levels. 

### 3.6. Bioinformatics and Statistical Analyses

The *lumi* module in the R-based *Bioconductor* package was used to transform the expression intensity to log2 scale. The log2 transformed intensity data were normalized using the Quantile normalization algorithm. The *Limma* program in the *Bioconductor* package under R computing environment was used to calculate the level of differential gene expression. Briefly, a linear model based on Bayesian method has been used to fit to the data (with cell means corresponding to the different conditions and a random effect for array). Each sample was analyzed in duplicate. For each comparison, we obtained the list of differentially expressed genes constrained by *p*-value < 0.05 and at least 1.2 fold change. Microarray and bioinformatics analysis were submitted to NCBI and the GEO accession number is GSE33918.

### 3.7. Real-Time PCR

Real-time PCR with forward and reverse primers and fluorescent probe 5’FAM and 3’TAMRA was performed, as described in [6]. The primers (marked f-for forward and r-for reverse) and fluorescent probes for microarray validation by RT-PCR were the following: RIP: f-5'-ATATCCCAGTGCCTGAGACC-3', r-5'-AGATTCATCTGTTGGTGGCA-3', and probe 5'-CCCAC CATGCCATTCAGCTCC-3'; Mdm-2: f-5'GAATCTACAGGGACGCCATC3', r-5'-CTGATCCAAC CAATCACCTG-3', and probe 5'-TCACTTACACCAGCATCAAGATCCGG-3'; PRSS8: f-5'-ATG GTGTGTGCTGGCTATGT-3', r-5'-CGTCAGGTACCAGAGACCCT-3', and probe 5'-CGCCTGCC AGGGTGACTCTG-3'; BRIP: f-5'-CAGATGAGGGCGTAAGTGAA-3', r-5'-TCTTTCAGAAGGT GGTGTGC-3', and probe 5'-TCATGTTGTTGTGCATGCCATTCA-3'; MET, f-5'-CAGCGCGTTGA CTTATTCAT-3', r-5'-CCCTCTGATGTCCCAAGATT-3' and probe 5'-CCACCTTCATTAAAGG AGACCTCACCA-3'; MTF, f-5'-CAATGCACTTCCACAACACA-3', r-5'-CCTGGGTCGTACTG GAATTT-3', and probe 5'-TTCTGTCCACAGATTCTGAATTGCGA-3'; PLK, f-5'-GAGCAGCTG AGCACATCATT-3', r-5'-CATGTGAGCACCATTGTTGA-3', and probe 5'-CAGACCACACCGTC GGTGTCC-3'; BIRC2, f-5'-GCTAGTCTGGGATCCACCTC-3', r-5'-AGAGGGTTTGGAGAAAG GCT-3', and probe 5'-TGCACATTCATTATCTCCCACCTTGG-3'. GAPDG was used as endogenous control, as described [6]. All experiments were performed in triplicates and RQ was calculated for each gene tested.

### 3.8. Statistical Analyses

Student’s t test was performed to determine significance. The difference between treated and untreated samples with *p* < 0.05 was considered significant.

## 4. Discussion

By microarray gene expression profiling analysis we identified differentially expressed genes in HCT116 p53^+^/^+^ cells affected by R2 in a p53-dependent manner. Among differentially expressed genes, important down-stream players of p53 signaling were identified, such as Mdm-2, Noxa-1, and RIP-1.

We detected 1484 genes that were up or down-regulated by R2 in HCT116p53^+/+^ cells but not in HCT116^−^^/−^ cells. Among these genes are Mdm-2, a known transcriptional target of p53 [16]. And it has been shown also to be up-regulated by Nutlins, which disrupted binding of Mdm-2 and p53 as a result of p53 increased activity and expression [16]. Among up-regulated genes, RIP-1 has been detected. In a previous report we demonstrated an association of FAK and RIP-1, where FAK sequestered pro-apoptotic RIP-1 from its pro-apoptotic functioning [17]. Thus, activation of RIP-1 by R2 can decrease the survival functions of colon cancer cells. Another gene, encoding Noxa-1 protein (Phorbol-12-myristate-13-acetate-induced protein 1) was one of the significantly up-regulated targets caused by R2. It is known that Noxa-1 is a transcriptional target of p53 and is a pro-apoptotic member of the Bcl-2 proteins family and is involved in p53-mediated apoptotic signaling [18].

Another up-regulated gene is BRIP1 (BRCA1-interacting protein 1) which encodes Fanconi anemia group J protein, a member of DNA helicase [19], known to be critical for breast cancer suppression and DNA repair. BRIP1 protein is mutated in the cancer prone syndrome Fanconi anemia, a disorder characterized by congenital malformations and a predisposition to the development of malignancies. At the molecular level, it is associated with chromosomal instability and defective DNA repair.

PRSS8 gene was also significantly up-regulated by R2. It encodes serine protease 8 or prostasin, known as a suppressor of invasion. Recently, loss of prostasin has been shown to be associated with epithelial-mesenchymal transition (EMT) [20].

Among down-regulated genes, Met gene encoding Met protooncogene has been detected. It is known that Met interacts with FAK and controls survival signaling in cancer cells [21]. Thus, the microarray study confirmed our functional data on up-regulation of p53 targets by R2 and revealed novel genes and signaling pathways affected by FAK and p53 disruption.

Another protein encoded by PLK2 (Polo-like Kinase 2), one of the p53 targets [22], was also affected by R2 [23]. Recently, PLK-2 deficient tumors demonstrated increased apoptosis in response to chemotherapy and silencing of PLK2 also caused increased apoptosis due to mitotic catastrophe, suggesting that PLK functions in the cell cycle arrest [24].

Among down-regulated proteins by R2 microarray analysis, we detected Mdm-2-binding protein, encoded by the MTBP gene. This protein has been shown to bind Mdm-2 [25] and regulates Mdm-2-dependent p53 homeostasis.

It is known that FAK and p53 interact and also that Mdm-2 interacts with p53 and with FAK [11]. Since, we detected increased Mdm-2 expression by R2, and Mdm-2 was shown to bind FAK and p53 to down-regulate p53 activity, we tested combination of drugs targeting of these complexes by combination of R2 and M13 or R2 and Nutlin-1. We demonstrated increased efficacy of the combinations in HCT116p53^+/+^ cells, but not HCTp53^−^^/−^ cells. Thus, the combination of therapies targeting FAK-p53 and FAK-Mdm-2 or FAK-p53 and Mdm-2-p53 can be important to enhance targeting of these complexes. Thus, Roslin 2 or R2 compound can be a potential approach for increasing p53 activity, similar to small molecule compounds such as Nutlins [16] or RITA [26] that are known to disrupt p53 and Mdm-2 interactions. The combination of FAK-p53 by R2 and FAK-Mdm-2 by M13 and Nutlin-1 targeting p53-Mdm-2 complexes can provide an effective combination therapy approach targeting the FAK scaffold [27] that can be used in future preclinical and clinical trials.

## 5. Conclusions

In conclusion, we demonstrated a gene expression profile in response to the small molecule compound R2 that targeted FAK and p53 interaction and showed that combination of FAK-p53-Mdm-2 targeting compounds that can be used as a novel approach *in vivo.* This study has a high impact on p53-regulated signaling and provides novel data on FAK and p53 interaction.
