# Correction: Golubovskaya et al. Gene Expression Profiling Identifies Important Genes Affected by R2 Compound Disrupting FAK and P53 Complex. *Cancers* 2014, *6*, 166–178

**DOI:** 10.3390/cancers18030526

**Published:** 2026-02-06

**Authors:** Vita M. Golubovskaya, Baotran Ho, Jeffrey Conroy, Song Liu, Dan Wang, William G. Cance

**Affiliations:** 1 Department of Surgical Oncology, Roswell Park Cancer Institute, Buffalo, NY 14263, USA; baotran.ho@roswellpark.org (B.H.); william.cance@roswellpark.org (W.G.C.); 2 Genomics Shared Resource, Center for Personalized Medicine, Roswell Park Cancer Institute, Buffalo, NY 14263, USA; jeffrey.conroy@roswellpark.org; 3 Bioinformatics Core Facility, Biostatistics, Roswell Park Cancer Institute, Buffalo, NY 14263, USA; song.liu@roswellpark.org (S.L.); dan.wang@roswellpark.org (D.W.)

**1.**  **Error in Figure**

In the original publication [1], there was a mistake in Figure 3A as published. The two colony images in the lower panel were similar. The corrected Figure 3A appears below.

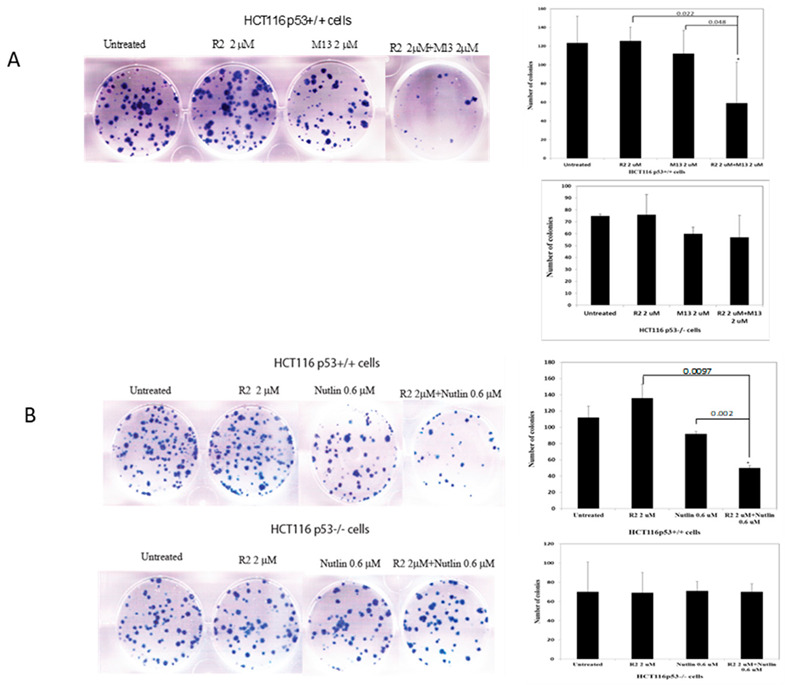

**2.**  **Update to Figure legend**

The corresponding Figure 3 legend has been updated to reflect the removal of some of the clonogenic assays.

**Figure 3.** R2 sensitized HCT116 cells to M13 and Nutlin. A clonogenicity assay was performed on HCT116p53^+^/^+^ cells or HCT116p53^−^/^−^ treated for one week either with R2 alone, M13 alone, or with a combination of R2 and M13 (**A**), and R2 alone, Nutlin-1 alone, or with a combination of R2 and Nutlin-1 (**B**). The representative clonogenicity assay of several independent experiments is shown. A clonogenicity assay image of HCT116p53^−^/^−^ in (**A**) is not shown. Colonies were counted and quantification is shown with the panels on the right. Bars show the average number of colonies per treatment from two to four independent experiments. * Student’s *t*-test, *p* < 0.05, R2 + M13 or R2 + Nutlin-1 versus untreated HCT116p53^+/+^ and HCT116 p53^−/−^ or treated cells with a single agent. The *p*-values are shown in a combination group versus a single agent.

**3.**  **Text Correction**

The text in Section 2.2 *The R2 Sensitized Cancer Cells to M13 (Disrupting FAK and Mdm-2) and Nutlin-1 (Disrupting p53 and Mdm-2) Treatments*, paragraph 2 has been updated to ‘Since Nutlin-1 that disrupts p53 and Mdm-2 protein interaction [15], also can induce p53, we treated HCT116 p53^+/+^ and HCT53^−/−^ cells with either R2 alone, Nutlin-1 alone or with combination of R2 and Nutlin-1 (Figure 3B)’.

The authors state that the scientific conclusions are unaffected. This correction was approved by the Academic Editor. The original publication has also been updated.
